# Improvement of Resistance to Clubroot Disease in the Ogura CMS Restorer Line R2163 of *Brassica napus*

**DOI:** 10.3390/plants11182413

**Published:** 2022-09-16

**Authors:** Jiao Chen, Jiahui Li, Mengya Ma, Bao Li, Yuanwei Zhou, Yongzhong Pan, Youjun Fan, Bin Yi, Jinxing Tu

**Affiliations:** 1 National Key Laboratory of Crop Genetic Improvement, Hubei Hongshan Laboratory, National Center of Rapeseed Improvement, Huazhong Agricultural University, Wuhan 430070, China; 2 Yichang Academy of Agricultural Science, Yichang 443009, China; 3 China Farm (Wuhan) Good-Seed Technology Company, Wuhan 430071, China

**Keywords:** *Brassica napus*, clubroot disease, Ogura CMS, MAS

## Abstract

Oilseed rape (*Brassica napus*) has significant heterosis and Ogura CMS is a major way to use it. Ogura CMS has the advantages of complete and stable male sterility and easy-to-breed maintainers. Therefore, to breed better restorers has become an important goal for this system. Incidentally, clubroot is a soil-borne disease that is difficult to control by fungicidal chemicals, and it has been the main disease of oilseed rape in recent years in China, severely restricting the development of the oilseed rape industry. At present, the most effective method for controlling clubroot disease is to cultivate resistant varieties. One Ogura CMS restorer line (R2163) has shown much better combining ability, but lacks the clubroot disease resistance. This study was carried out to improve R2163 through marker-assisted backcross breeding (MABB). The resistant locus *PbBa8.1* was introduced into the restorer R2163, and we then selected R2163R with clubroot disease resistance. Using the new restorer R2163R as the male parent and the sterile lines 116A and Z11A as the female parent, the improved, new resistant hybrids Kenyouza 741R and Huayouza 706R performed well, providing strong resistance and good agronomic traits. This work advances the utilization of heterosis and breeding for clubroot disease resistance in *B. napus*.

## 1. Introduction

Oilseed rape is one of the most important oilseed crops in the world. It is also one of the fastest-improving crops in recent decades regarding utilization of heterosis to increase production. The yield of oilseed rape hybrids can be increased by more than 20% compared to open-pollinated varieties, with an increase in the oil content [1]. Ogura cytoplasmic male sterility (CMS) is an important way to utilize the heterosis of oilseed rape. It has the advantages of complete male sterility and is unaffected by the ambient temperature, and male sterile lines are easy to breed because any lines could be as its maintainers. Currently, breeding of Ogura CMS restorer lines is one of the main research priorities in the development of heterosis. Therefore, the establishment of the Ogura CMS restorer system has also become an important goal.

In 1968, Ogura discovered the natural radish cytoplasmic male sterile line Ogura CMS in the Japanese radish population [2]. Its sterility was stable and thorough, but the restoration source of the Ogura CMS line did not exist in oilseed rape varieties. Therefore, the main challenge facing heterosis utilization in *B. napus* is to select the Ogura CMS restorer line. The nucleus of *B. napus* was introduced into the sterile cytoplasm of Japanese radish through interspecific hybridization and backcrossing to obtain the *B. napus* Ogura CMS [3]. The nuclear gene *Rfo* can repair the CMS of radish in *B. napus* by changing the translation and expression of the mitochondrial gene *orf138* [4]. Protoplast fusion technology can make *B. napus* material obtain the ability to completely restore the fertility of Ogura CMS [5]. However, when the restorer gene was introduced into *B. napus*, the high glucosinolate gene and the redundant fragments closely linked to the restorer gene were also introduced, resulting in poor agronomic characteristics of the restorer line and rendering its use in commercial production challenging [6].

Until 2000, the French Institut national de la recherche agronomique (INRA) used *γ*-ray irradiation to induce recombination to cultivate a restorer line R2000 with low glucosinolate content and good fertility recovery ability [7]. After introducing the Ogura restore locus from Indian mustard into oilseed rape, a new restorer line R206 was obtained [8]. Compared to the restorer line R2000, R206 has a shorter linkage fragment of the radish chromosome, and the restore gene has a specific marker RMC16 located in chromosome A10, not in chromosome C09. Breeding new and excellent Ogura CMS restorer lines could provide more approaches and resources for the utilization of heterosis in *B. napus*.

Clubroot is a soil-borne disease seriously affecting oilseed rape roots, which is caused by the protist pathogen *Plasmodiophora brassicae* Woronin [9]. In production, the incidence of clubroot disease is even higher than 15% in many places, and traditional agricultural control is largely unsuccessful [10]. The dormant spores can survive in the soil for more than 10 years after leaving the host, causing the field to carry the pathogen for a long time and facilitating rapid spread of the disease [11]. Therefore, it is difficult to control and prevent infection through fungicides and crop rotation. In addition, flowing water, agricultural operations, and transportation of diseased seedlings facilitate the spread of clubroot disease [12,13].

It is difficult to achieve good control using cultural methods, and many production challenges need to be addressed [14,15]. In addition, chemical control, biological control, and cultivation of resistant varieties can be used to prevent and control *P. brassicae*, but breeding for resistant varieties is the most economically feasible measure. The discovery of clubroot-resistant varieties provides beneficial resources for disease resistance breeding. Studies have shown that European forage turnip (*Brassica rapa ssp. rapifera* L, AA, 2n = 20) has the most disease-resistant sites, while oilseed rape has relatively few disease-resistant resources. After the researchers identified 955 resources, only 4 highly resistant rapeseed materials were found, and the results showed that disease-resistant or immune resources mainly exist in *B. rapa* [16]. In addition, ECD01-04 in the European clubroot identification system (ECD) has extensive resistance to clubroot, and ECD06, ECD08, ECD09, and ECD10 also show resistance to some physiological races [17]. The disease resistance sites in turnip (ECD04) were introduced into *B. napus*, and the clubroot-disease-resistant variety Mendel was finally bred, after which the clubroot disease resistance genes in Mendel were used to develop more clubroot resistant varieties [18]. The resistance genes of *B. napus* that have been discovered are mainly derived from the A genome, and more than 20 resistance quantitative trait loci (QTL) have been found in *B. napus* through genome-wide association analysis [19,20].

In recent years, new sources of resistance such as *Rcr2*, *Rcr5*, *Rcr3*, and *Rcr9wa* have been discovered in Chinese cabbage [21,22,23], as well as the new sites *CRs* QTL and *PbBrA08^Banglim^* [24,25]. Clubroot resistance (CR) resources have also been found in other Brassicaceae, such as the *Crs1* locus in radish (*Raphanus sativus* L., RR) and *Rcr6 in Brassica nigra* (L.) W.D.J.Koch (AABB) [26,27]. CR genes that are highly resistant to clubroot disease exist in European forage turnips, such as the *Cra* gene located in ED02 [28,29], and the *CRb* gene located on the A3 chromosome, which is resistant to races 2, 4, and 8 [30]. Further studies have shown that *Cra* and *CRb* are the same gene [31]. In Chinese cabbage, the *Crr1*, *Crr2*, and *Crr4* genes are located on chromosomes A08, A01, and A06, respectively [32,33]. The *Crr3* gene is located in turnip varieties [34]. The *CRk* and *CRc* sites are close to *Crr3* and are located on chromosomes A3 and A2, respectively, while *CRd* is located upstream of *Crr3* [35,36]. *PbBa1.1* is located on the A1 chromosome, *PbBa3.1*, *PbBa3.2*, and *PbBa3.3* are located on the A3 chromosome, and *PbBa8.1* is located on chromosome A8 [37]. *Rcr1*, *Rcr2*, and *Rcr4* are located on the A3 chromosome, *Rcr9* and *Rcr3* are located on the A8 chromosome, and *Rcr8* is located on the A2 chromosome [21,23,38,39]. The identification of clubroot-disease-resistant sites and the development of related molecular markers have advanced the process of molecular-marker-assisted selection breeding.

Introducing resistance genes into oilseed rape to breed varieties with clubroot disease resistance is the most economical and effective way to prevent clubroot disease. In this study, Huashuang 5R, with the resistant locus *PbBa8.1*, was used as the donor parent, and the Ogura CMS restorer line (R2163) without clubroot disease resistance was used as the recurrent parent. The *PbBa8.1* site in Huashuang 5R was introduced into R2163, and the R2163R with clubroot disease resistance was selected by MABB. Finally, two new hybrids, Kenyouza 741R and Huayouza 706R, with clubroot disease resistance were bred. The combination of heterosis breeding, disease resistance breeding, and the use of MABB will promote the utilization of heterosis and improve the efficient development of the oilseed rape industry.

## 2. Results

### 2.1. Marker-Assisted Introgression of PbBa8.1 into R2163

The restorer line R2163 was crossed with Huashuang 5R, and the “true” F_1_ plants were identified by the molecular markers A08-300, cnu_m090a, and sau_um353a, closely linked to *PbBa8.1* [40], and the molecular marker BnRFO-AS2, closely linked to the Ogura fertility restorer gene *Rfo* [41]. They were then backcrossed with R2163. Foreground selection analysis was performed on 72 individual plants of the BC_1_ population, and these individual plants were identified and screened using molecular markers (A08-300, cnu_m090a, sau_um353a, and BnRFO-AS2) (Table S1). The results showed that 32 plants contained the Ogura fertility restorer gene *Rfo* and the *PbBa8.1* loci (Table S2).

**Figure 1:**
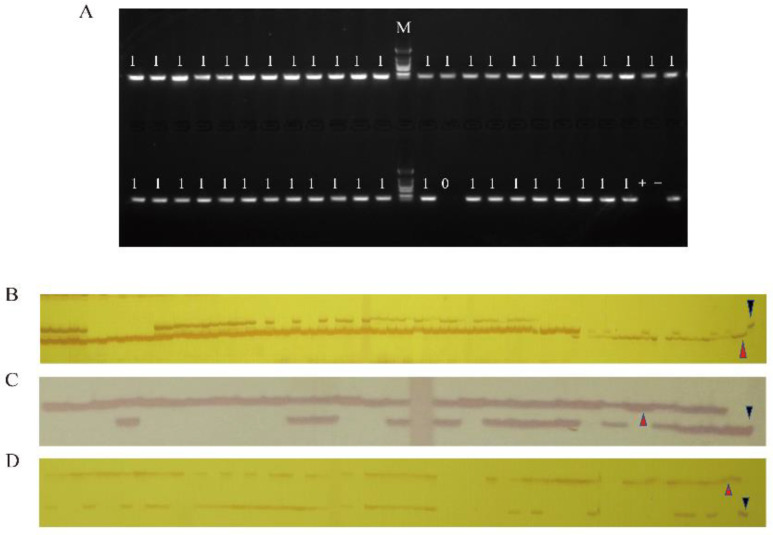
Identification results of prospect selection in the BC_1_ population. The images show the identification results of the four markers. (**A**) Polymorphism detection of marker BnRFO-AS2, 0: No specific band has been amplified; 1: Specific band has been amplified; M: Marker. (**B**) Polymorphism detection of marker cnu_m090a. (**C**) Polymorphism detection of marker A08-300. (**D**) Polymorphism detection of marker Sau_um353. Red triangles represent R2163 without resistance and blue triangles represent Huashuang 5R with resistance.

Some of the results of the resistance identification and restoration gene identification are shown in Figure 1. From the identified individual plants containing both the *Rfo* gene and the *PbBa8.1* loci, 20 plants were selected to backcross with R2163 to obtain the BC_2_ population. Foreground selection analysis was performed on 40 individual plants of the BC_2_ population, and they were identified and screened using molecular markers. The results showed that 21 plants contained the Ogura fertility restorer gene *Rfo* and the *PbBa8.1* loci (Table S2)

**Figure 2:**
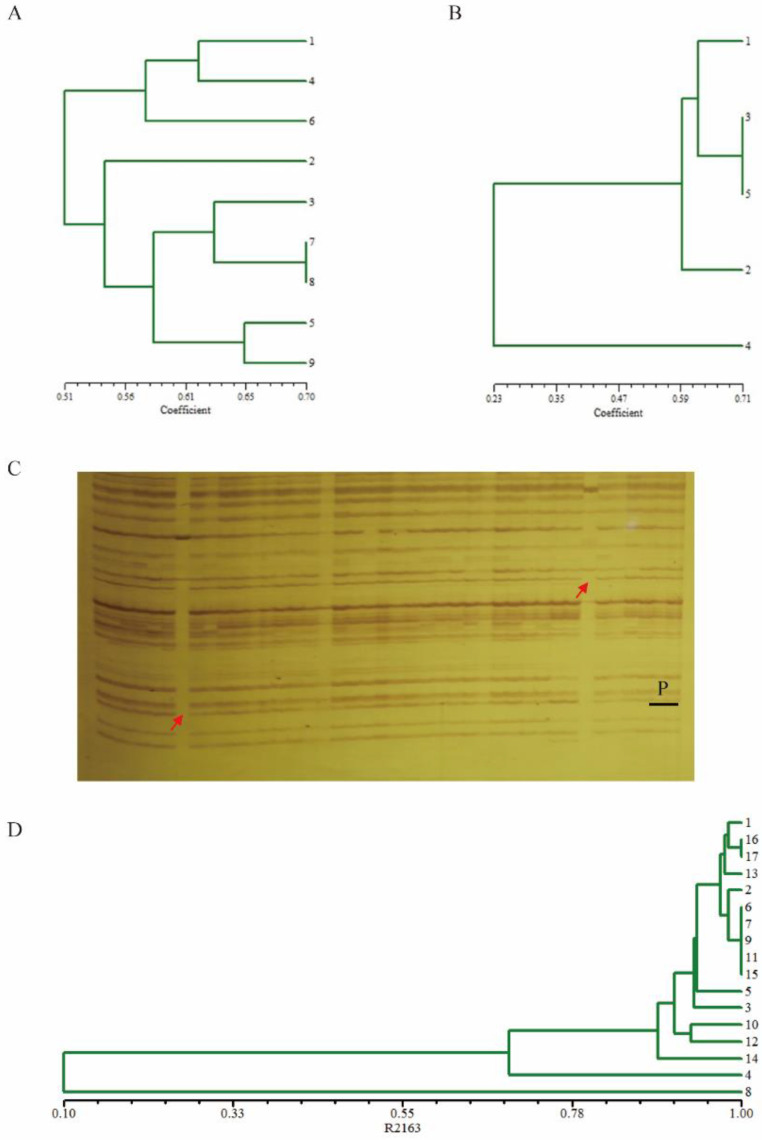
Background selection for BC_2_, BC_3_, and BC_4_ populations. (**A**) The UPGMA cluster graph of the recurrent parent and BC_2_ individuals, where 9 represents the recurrent parent R2163 and 1–8 represent individuals of the BC_2_ population. (**B**) The UPGMA cluster graph of the recurrent parent and BC_3_ population, where 5 represents the recurrent parent R2163, 4 represents the non-recurrent parent, and 1–3 represent individuals of the BC_3_ population. (**C**) Background screening of individuals in the BC_4_ population using AFLP primer, P: Recurrent parent R2163; the others are individuals containing the restorer gene and resistance gene in the BC_4_ population; the arrows indicate polymorphic fragments. (**D**) The UPGMA cluster graph of the recurrent parent R2163 and BC_4_ individuals; 16–17 represent the recurrent parent R2163, 1–15 represent individuals of the BC_4_ population.

**Figure 3:**
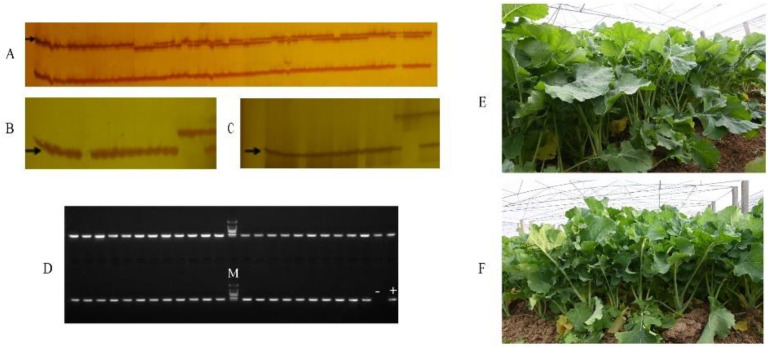
Polymorphism detection of markers in the BC_4_F_2_ population. (**A**) Polymorphism detection of marker cnu_m090a. (**B**) Polymorphism detection of marker A08-300. (**C**) Polymorphism detection of marker sau_um353. Arrows indicate bands with homozygous resistance genotypes. (**D**) Polymorphism detection of marker BnRFO-AS2; the picture shows the identification results of lines that are homozygous for the restorer gene and do not segregate. (**E**) Field phenotypic investigation of R2163. (**F**) Field phenotype investigation of the restorer line R2163R with clubroot disease resistance after improvement.

On the basis of the foreground selection results, background selection was carried out using the *Sac*I/*Mse*I restriction endonuclease digestion combination, pre-amplification primer selection with the SA/MG combination, and 20-60 pairs of selected amplification primers for PAGE gel electrophoresis detection. After finding the polymorphic bands and counting the results, four plants were identified with a relatively high PRPG (Proportion of Recurrent Parent Genome) of 80.23%, 83.14%, 80.23%, and 76.16%, respectively (Table S3, Figure 2A). The individual plants with a relatively high PRPG in the BC_2_ population were used for the continuation of backcrossing with R2163 to obtain the BC_3_ population. A total of 40 individual plants of two BC_3_ populations were then identified by molecular markers to determine whether they contained clubroot resistance genes and the *Rfo* gene. Finally, 20 individual plants were found to simultaneously possess both target genes (Table S2). Background selection was carried out using the *Taq*I/*Pst*I restriction endonuclease digestion combination, and the P0/TC combination was used for pre-amplification primer selection. We used 20–60 pairs of primers for the selected amplification and found 223 polymorphic bands. The results showed that the background recovery rate of the progeny of the BC_3_ generation had reached 88.60% (Table S3, Figure 2B). BC_3_ continued to backcross with R2163 to yield the BC4 population. There were 42 individual plants of three BC_4_ populations that were screened by molecular markers, and the identification results showed that 16 plants contained the Ogura fertility restorer gene *Rfo* and the clubroot resistance locus *PbBa8.1* (Table S2). On the basis of the foreground selection, the *Eco*RI/*Taq*I restriction endonuclease digestion combination was used for background selection (Figure 2C), using the pre-amplification primer selection combination TC/EA. The results showed that the PRPG of seven individual plants of the BC_4_ population had reached more than 99% (Table S3, Figure 2D), indicating that the background of these individual plants was very close to the recurrent parent. The results of the background selection of individual plants in each generation are listed in Table S3. There were seven individual plants to be selfed, from which the BC_4_F_2_ population was derived, and then 138 plants of the BC_4_F_2_ population were identified by molecular markers. We found 70 individual plants that met our expected target, i.e., containing both target genes simultaneously (Table S2). The BC_4_F_3_ generation was obtained by selfing the plants from the BC_4_F_2_ progeny with both the homozygous clubroot resistance gene and the homozygous restorer gene (Figure 3A–D). The harvested BC_4_F_3_ generation seeds were planted in Wuhan, and foreground selection was performed at the seedling stage. The BC_4_F_3_ line with both homozygous clubroot resistance genes and homozygous restorer genes was regarded as an improved, genetically stable line. The improved Ogura CMS restore lines with clubroot resistance were called R2163R. We conducted field observations on the restorer line R2163 before improvement and the restorer line R2163R after improvement. The results showed that the individual plants of the improved restorer line were similar to the restorer line R2163 in appearance, such as leaf color and shape, indicating that the genetic backgrounds of R2163 and R2163R were already very similar (Figure 3E,F).

### 2.2. Preparation and Clubroot Resistance Identification of the Hybrids Kenyouza 741R and Huayouza 706R

**Figure 4:**
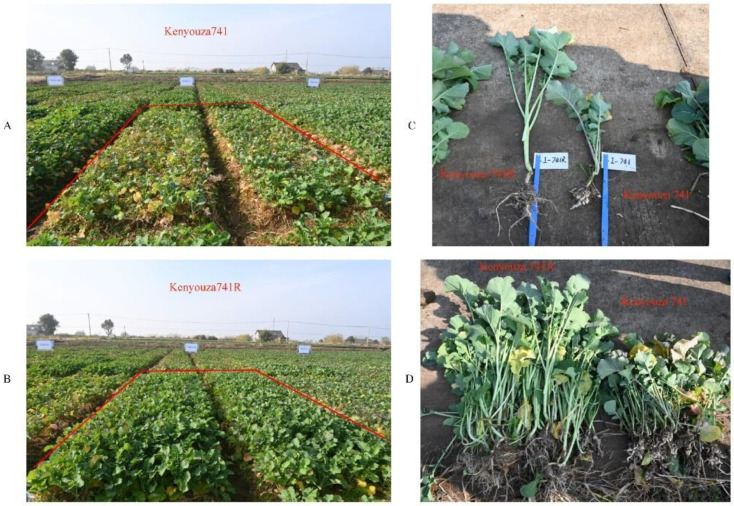
Investigation of clubroot resistance in Kenyouza 741R and Kenyouza 741. (**A**,**B**) Field phenotypic investigation of Kenyouza 741 and Kenyouza 741R. The observation area is shown in the red line frame of (**A**,**B**). (**C**,**D**) Comparison of root phenotype investigation of Kenyouza 741R and Kenyouza 741.

**Figure 5:**
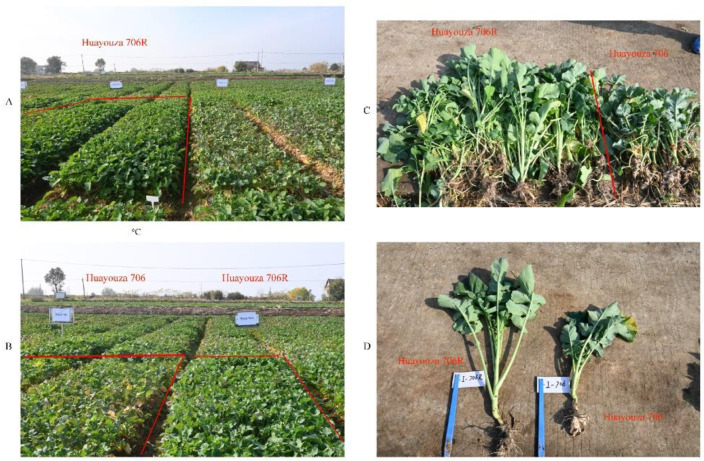
Investigation of clubroot resistance in Huayouza 706R and Huayouza 706. (**A**,**B**) Field phenotypic investigation of Huayouza 706R and Huayouza 706. The observation area is shown in the red line frame of (**A**,**B**). (**C**,**D**) Comparison of root phenotype investigation of Huayouza 706R and Huayouza 706.

To further apply R2163R to production, it was subjected to phenotypic evaluation for disease resistance. The restorer line material R2163R was used as the male parent, and the sterile line materials 116A and Z11A were used as the female parent, respectively, and the new, improved clubroot-resistant varieties Kenyouza 741R and Huayouza 706R were obtained. In the seedling stage, Kenyouza 741R and Huayouza 706R were investigated for resistance to clubroot disease in the field, and they were found to have strong disease resistance and good field growth (Figure 4A–D and Figure 5A–D). In the planting areas of Kenyouza 741 and Huayouza 706 without resistance, the plants grew weakly, and the leaves turned yellow and withered (Figure 4A–D and Figure 5A–D).

### 2.3. Clubroot Resistance Evaluation

In order to evaluate the resistance of Kenyouza 741R and Huayouza 706R, we randomly selected three sampling areas in each plot, surveyed 1 m^2^ at each area, counted the total number of plants and the number of susceptible plants, and calculated the disease incidence. We regarded the plants with obviously swollen roots as susceptible. A total of 384 plants of Kenyouza 741R were investigated, of which 21 were susceptible, with a disease incidence of 5.47%. In contrast, a total of 393 plants of Kenyouza 741 were investigated in the control group, all of which displayed clubroot symptoms, for a disease incidence of 100%. A total of 372 plants of Huayouza 706R were investigated, and 3 were susceptible, with a disease incidence of 0.81%, while in the control group, all 365 Huayouza 706 plants were susceptible (100% disease incidence) (Table 1). The survey results showed that Kenyouza 741R and Huayouza 706R had higher resistance compared to the control, which indicated that the improved restorer line R2163R had higher resistance and could be used in hybrid seed production.

### 2.4. Evaluation of Agro-Morphological Characters of the Improved Kenyouza 741R and Huayouza 706R

**Figure 6:**
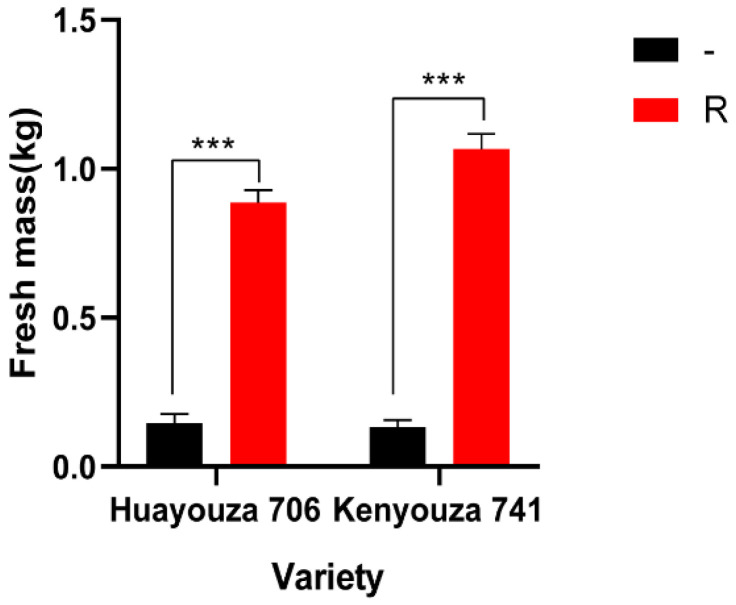
Evaluation of agro-morphological characters of the improved Kenyouza 741R and Huayouza 706R. -: Huayouza 706/Kenyouza 741; R: Huayouza 706R/Kenyouza 741R. *** Significance: *p* < 0.01.

To better evaluate the agronomic traits of the cultivars with clubroot disease resistance, we weighed their aerial parts at the seedling stage. The results showed that the fresh mass of 20 plants of Kenyouza 741R was 1.07 kg, while that of Kenyouza 741 was 0.13 kg, and the fresh mass of Huayouza 706R was 0.89 kg, while that of Huayouza 706 was 0.15 (Table 2). Kenyouza 741R and Huayouza 706R were significantly heavier than the non-resistant varieties Kenyouza 741 and Huayouza 706 (Table 2, Figure 6). This showed that the non-resistant varieties had been seriously damaged by clubroot at the seedling stage, which affected their normal growth, while the resistant varieties showed strong resistance, and the seedling stage showed superior size and quality.

## 3. Discussion

The sterility of Ogura CMS is stable and complete, but there is no restorer source in *B. napus*. Scientists have achieved the transfer of the radish restoration gene into *B. napus* through protoplast fusion and ray induction [5,7]. However, the Ogura restorer gene also carries unfavorable traits, such as high glucosinolate content, poor seed set, and weak cold resistance, which leads to poor agronomic and quality traits in the selected *B. napus* Ogura CMS restorer lines that cannot meet production requirements [42,43]. The breeding and improvement of the Ogura CMS restorer line of *B. napus* in China is still in an early developmental stage, and an effective method for shortening the radish genome segment has not yet been found [44].

Breeding *B. napus* Ogura CMS restorer lines has become most challenging aspect for the utilization of heterosis. In the present study, we found that the Ogura CMS restorer gene (*Rfo*) was genetically unstable, and its performance was inconsistent with Mendelian inheritance, which indicated that the radish chromosome segment had a significant impact on fertility segregation, resulting in a longer time for the selection of restorer lines and a higher number of generations. Similarly, some researchers investigated the fertility segregation ratio of 201 inbred lines (including fertile lines of backcross progeny) and found that the lines that reached or exceeded the 3:1 fertility segregation ratio only accounted for 6% of the total selection coefficient [45].

The radish chromosome segment linked with the restorer gene has brought great difficulties to the breeding and improvement of Ogura CMS restorer lines. It is difficult to break the linkage relationship using conventional breeding methods, and it is usually only possible to break it by combination with modern biotechnologies such as molecular-marker-assisted selection and microspore culture [46]. Therefore, using MAS (molecular-marker-assisted selection) to breed an excellent Ogura CMS restorer line of *B. napus* is conducive to the utilization of male sterility in Ogura CMS. The restorer line R2163 used in this study comes from a recurrent selection population with C subgenome diversity [47]. The restorer line that was crossed with a sterile line with a Chinese rapeseed background had high combining ability and good breeding prospects. Therefore, we further improved the resistance of R2163 with excellent agronomic traits, in order to obtain restorer lines with strong clubroot disease resistance (R2163R) that met the needs of the oilseed rape industry.

At present, breeding disease-resistant varieties is the most economical and feasible measure to address clubroot disease challenges. The resistance loci of clubroot disease were directionally introduced into *B. napus*, and new varieties that met the requirements of breeders could be obtained quickly and with reduced effort by MABB. In addition, the distribution of *P. rhizogenes* in China is dominated by race 4 [48]. The *PbBa8.1* locus introduced into R2163 in this study specifically targets race 4. Therefore, the resistance improvement of R2163 will be used in the production and utilization of oilseed rape in the future. Multiple resistance loci can be further aggregated so that they can act on a variety of physiological races and better serve oilseed rape production.

Using R2163R, we bred the new disease-resistant varieties Kenyouza 741R and Huayouza 706R, and their performance in the field was far better than that of the controls (Figure 4 and Figure 5). The non-resistant varieties Huayouza 706 and Kenyouza 741 both had 100% disease incidence at the seedling stage (Table 1), and the fresh weight of above-ground biomass was significantly lower than that of Huayouza 706R and Kenyouza 741R (Table 2, Figure 6), indicating that the effect of resistance improvement was significant. However, we found that new varieties introduced with other resistance loci were less resistant than Huayouza 706R and Kenyouza 741R in this area, but showed better resistance in other areas, indicating that the dominant physiological races in different places may be different and the physiological races in the same place will also change over time. Therefore, in order to better prevent clubroot disease in the future, aggregation breeding is the current focus of attention. At present, there are only a few varieties of rapeseed resistant to clubroot in China. Our work provides new materials for oilseed rape production and utilization, and also promotes the development of a restorer line R2163R in production and application.

MAS technology not only speeds up the breeding process to a large extent, but also improves accuracy and is a more effective method than phenotypic selection. It is very important for hybrid breeding and commercial breeding, and it can be used to assist gene infiltration to interpenetrate genes of different elite germplasm materials to achieve the purpose of character complementation [49]. MAS can also assist in gene aggregation, i.e., in aggregating multiple favorable genes/loci that control traits to maximize their utilization [50].

In our study, using MABB, the clubroot disease resistance site (*PbBa8.1*) was introduced into the restorer line (R2163). In the breeding process, we did not carry out the identification of the resistance phenotype in each generation, but only carried out MAS. However, the resistance performance of the final hybrid combination was very good. This shows that, under the premise of reliable molecular markers, only MAS can achieve the expected goals. In this study, the foreground selection was performed on the individual plants in each generation of the population, and on this basis, the background selection was performed, which accelerated the breeding process. Therefore, we provided a feasible method for breeding excellent restorer lines with clubroot disease resistance based on the Ogura CMS restorer line in *B. napus*. Prospect selection in this study included identification of the radish restorer gene *Rfo* and identification of the clubroot resistance gene, and our simultaneous selection of multiple pairs of molecular markers that could improve the accuracy of selection. Combined with backcross breeding, after multi-generation backcross and self-crossing, two target gene homozygous progeny individual plants appeared in the BC_4_F_2_ population, laying a solid foundation for subsequent experiments.

In this study, background selection was performed by AFLP technology, which greatly improved the background recovery efficiency. The main purpose of background selection is to reduce the linkage burden and restore the recurrent parental genome as quickly as possible. In this study, different combinations of enzyme digestion were selected in different backcross progeny groups for screening, which can effectively improve the selection efficiency. At the BC_4_ generation, the background recovery rate of plants in the population was as high as 99%.

During the phenotypic investigation of the improved progeny, it was found that they were morphologically highly similar to the recurrent parents (Figure 3E,F), which further verified the reliability of the results. With the increase in generations, the background recovery rate of individual plants in the population continued to increase, which indicated that the genetic background of the offspring and the recurrent parents after backcrossing became more and more consistent. In theory, the background recovery rate of plants in the BC_3_ generation population is about 93.75%. However, the background recovery rate actually achieved in this study was lower than the theoretical value, and the possible reason was that the genetic background was detected on the premise of ensuring that the plants contained disease resistance genes and recovery genes, so there was a deviation from the theoretical value.

When using molecular-marker-assisted selection, we need to consider the number of backcrosses, population size, the number of target genes, the number and distance of molecular markers, etc. According to the results of computer simulations, the research shows that the key factor affecting MAS efficiency is population size, i.e., the larger the population, the higher the relative efficiency of the MAS [51,52,53]. Therefore, when more target genes are involved, expanding the backcross population ensures that there are enough individuals containing the target gene for background selection, and the more individuals used for background selection, the greater the degree of accuracy. In addition, molecular aggregation breeding of multiple resistance loci is crucial for resistance breeding for oilseed rape clubroot in the future.

In summary, Huashuang 5R was used as the donor parent, Ogura CMS restorer lines (R2163) without clubroot disease resistance sites were used as the acceptor parent, and the *PbBa8.1* locus was introduced into R2163. The restorer line R2163R with clubroot resistance was selected by MABB technology. Using R2163R, the new disease-resistant varieties Kenyouza 741R and Huayouza 706R were developed. In the future, the development of oilseed rape mechanization will not only improve productivity, but also bring great challenges to the prevention and control of oilseed rape clubroot disease. Currently, there are very limited disease-resistant varieties on the market that are suitable for planting in different ecological areas. Therefore, the genetic improvement of resistance to clubroot should be the focus of future prevention and control of clubroot.

## 4. Materials and Methods

### 4.1. Plant Materials and Technical Route of R2163R Breeding

**Figure 7:**
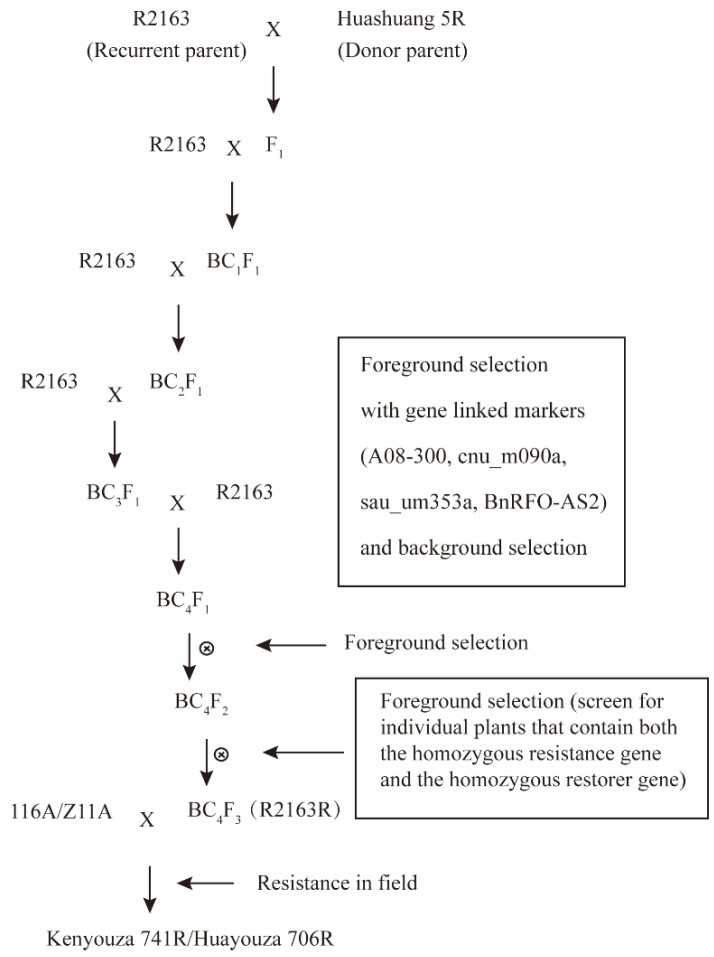
Scheme for the development of *PbBa8.1* loci into R2163.

The *B. napus* Ogura CMS restorer line R2163 has no clubroot disease resistance, and Huashuang 5R has clubroot disease resistance at site *PbBa8.1*. The male parent R2163 was obtained by selfing the double-low line 447 from the third generation of the population, which was composed of male sterile plants of the R recurrent selection population × R206. The R recurrent selection population included 108 F_6_ lines from the crosses [3 European winter oilseed rape lines × artificially synthesized *B. napus* (41 lines)], seven subgenome materials, two Polima restorer lines, etc. The restorer gene was used as a random mating system in the population. Huashuang 5R was provided by Professor Zhang Chunyu from Huazhong Agricultural University. F_1_ was obtained by crossing Huashuang 5R as the male parent (donor parent) and R2163 as the female parent (recipient parent). The F_1_ and R2163 populations were backcrossed to obtain the BC_1_ population, after which foreground and background selection on the offspring of the BC_1_ population was carried out. The plants with the clubroot-disease-resistant gene and restorer gene with relatively high background recovery rate were selected and crossed with R2163 to harvest the BC_2_ generation seed. The BC_3_ and BC_4_ populations were obtained by continuous backcrossing, and foreground selection and background selection were performed in each generation to select individual plants with *PbBa8.1* and *Rfo* for further research. The individual plants for which the background recovery rate was as high as 99% were self-bred to obtain the BC_4_F_2_ generation. BC_4_F_2_ generation seeds were planted, prospect selection was carried out at the seedling stage, and plants with homozygous clubroot disease resistance genes and homozygous restorer genes were selected for self-breeding to obtain the BC_4_F_3_ generation. Prospect selection was conducted on plants from the BC_4_F_3_ generation at the seedling stage, and the improved R2163R individuals that were genetically stable and resistant to clubroot disease were screened. The breeding process is shown in Figure 7.

### 4.2. Preparation of Hybrids of Kenyouza 741R and Huayouza 706R

The restorer line R2163R with clubroot disease resistance as the male parent and the male sterile lines 116A and Z11A as the female parent were used to obtain the improved hybrids Kenyouza 741R and Huayouza 706R with clubroot disease resistance. The maintainer line 116B was derived from the progeny of the individual plant No. 116 in the maintainer recurrent selection population, and the maintainer line Z11B was also from the individual plant No. 11 in the same recurrent selection population. The maintainer recurrent selection population was composited with 30 open-pollination varieties from China, Dunkeld from Australia, Q2 from Canada, and some Polima CMS maintainer lines. The dominant GMS gene was introduced to obtain the maintainer recurrent selection population as a random male pollination system.

### 4.3. Evaluation of Clubroot Resistance in Kenyouza 741R and Huayouza 706R

The experiment was arranged in the Zhijiang Experimental Base of Yichang Agricultural Science Research Institute, Yichang, China. A random block arrangement with two repetitions and a plot area of 20 m^2^ was used in the study. The previous crop was rice, and the oilseed rape seeds were sown on 26 September 2021. The seeding rate was 500 g/ac, and 40 kg/ac of the special fertilizer for oilseed rape was used. After sowing, unified field management was adopted. At the seedling stage, Kenyouza 741R and Huayouza 706R were evaluated for clubroot disease resistance in the nursery. Three sampling points were randomly selected in each plot, and each point in a 1 m^2^ subplot was evaluated. Finally, the total number of plants and the number of diseased plants in each subplot were counted to calculate the clubroot disease incidence rate.

### 4.4. Foreground and Background Selection

The prospect options for this study included identification of the clubroot resistance loci *PbBa8.1* and *Rfo* gene. The resistance site *PbBa8.1* in Huashuang 5R was introduced into R2163, and three pairs of molecular markers (A08-300, cnu_m090a, and sau_um353a) closely linked to the *PbBa8.1* site were used to identify the clubroot disease resistance site, while the molecular marker BnRFO-AS2 (closely linked with the *Rfo*) was used to identify the existence of the gene [40,41]. AFLP technology was used for background selection, different enzyme digestion combinations were used for each generation, selective amplification primers were randomly selected for identification, and selective amplification products were detected by polyacrylamide gel electrophoresis. Statistical analysis was performed on the band pattern with polymorphism, and the Proportion of Recurrent Parent Genome (PRPG) was calculated [54]. The obtained data were clustered using NTsys 2.1 and a cluster diagram was constructed. The AFLP linker and primer sequences were designed according to the method reported by [55]. The primer sequences used for foreground and background selection are detailed in Tables S1 and S4.

### 4.5. DNA Extraction and PCR System

The CTAB method was used to extract DNA from fresh young leaves at the seedling stage [56]. For foreground selection, SCAR molecular marker PCR parameters were: 94 °C for 5 min; 34 cycles of 94 °C for 30 s, 55 °C for 30 s, and 72 °C for 30 s; 72 °C for 10 min, and 25 °C for 10 min. The SCAR molecular marker PCR reaction system is shown in Table S5. The PCR parameters for SSR and Indel molecular markers were as follows: 94 °C for 5 min; 94 °C for 45 s, 60 °C for 30 s, and 72 °C for 45 s, after which the annealing temperature was reduced by 0.5 °C for each cycle, for a total of nine cycles; 94 °C for 45 s, 55 °C for 30 s, and 72 °C for 45 s, for a total of 29 cycles; 72 °C for 10 min and 25 °C for 30 min.

For background selection, the PCR reaction parameters for pre-amplification were 94 °C for 5 min; 94 °C for 30 s, 56 °C for 30 s, and 72 °C for 1 min, for 20 cycles. The pre-amplification product was diluted to an appropriate multiple and mixed as a template for selective amplification. The PCR reaction parameters for selective amplification were: 94 °C for 5 min; 94 °C for 30 s, 65 °C for 30 s, and 72 °C for 1 min, and then the annealing temperature for each cycle was decreased by 0.7 °C during a run of 13 cycles, and then 23 cycles at 94 °C for 30 s, 56 °C for 30 s, and 72 °C for 1 min were run.

### 4.6. Evaluation of Agro-Morphological Characters of the Improved Kenyouza 741R and Huayouza 706r

A total of three biological replicates were set up in the experiment. Sampling points were randomly selected on the basis of avoiding marginal positions in each plot, and then 20 plants were pulled continuously. Finally, the roots below the stem nodes were removed and the above-ground biomass was weighed.

## Figures and Tables

**Table 1:** Clubroot resistance evaluation of Kenyouza 741R and Huayouza 706R.

Variety	Number of Diseased Plants	Total Number of Plants Surveyed	Disease Incidence %
**Kenyouza 741**	393	393	100.00
**Kenyouza 741R**	21	384	5.47
**Huayouza 706**	365	365	100.00
**Huayouza 706R**	3	372	0.81

**Table 2:** Fresh mass of the aerial parts of Kenyouza 741R and Huayouza 706R plants from replicates I to III.

Variety	Fresh Mass of Aerial Parts of 20 Plants (kg)				*p*-Value
	Ⅰ	Ⅱ	Ⅲ	Average Value	
**Kenyouza 741**	0.16	0.12	0.12	0.13	0.000016
**Kenyouza 741R**	1.12	1.06	1.02	1.07	
**Huayouza 706**	0.14	0.12	0.18	0.15	0.000008
**Huayouza 706R**	0.92	0.90	0.84	0.89	

Significance: *p* < 0.05.

## Data Availability

The data that support the findings of this study are available in the Supplementary Materials of this article.

## Supplementary Materials

The following supporting information can be downloaded at: https://www.mdpi.com/article/10.3390/plants11182413/s1, Table S1: Primers used in foreground selection; Table S2: Result statistics of prospect selection; Table S3: Result statistics of background selection; Table S4: Primers used in background selection; Table S5: Reaction systems for PCR amplifications.
