# Supplementary material for: Improvement of Resistance to Clubroot Disease in the Ogura CMS Restorer Line R2163 of Brassica napus
Source: Plants (Basel). 2022 Sep 16;11(18):2413. doi: 10.3390/plants11182413 (PMC9504965; doi:10.3390/plants11182413)
Supplement: Supplementary file 1 [file plants-11-02413-s001.zip › plants-1881919-supplementary.pdf]

Table S1. Primers used in foreground selection

Primer	Sequences
**A08-300F**	GTAGTGCGGGCCACAAAAT
**A08-300R**	CACAATGGAGTGTTGAAATTCACT
**cnu_m090a F**	GCAAAGATCGGCGAAGAAGA
**cnu_m090a R**	TGCAGACACATTCGAACAAACA
**sau_um353a F**	CCCACCTCCTGCTGATTGTA
**sau_um353a R**	GATTGATCTCCGGTGATTGG
**BnRFO-AS2F**	CATGCTTCGATCTCGTCCTTTA
**BnRFO-AS2R**	GGTAACAACATCAGGGTGGAGT

Table S2. Result statistics of prospect selection

Population	Total number of samples	Individual plant numbers with resistance and *Rfo* gene
BC_1	12	2, 5, 7, 8, 11, 12
BC_1	12	1~5, 7, 8, 10
BC_1	12	2, 7~12
BC_1	12	4, 5, 12
BC_1	12	7, 9
BC_1	12	3, 4, 6, 8, 11, 12
BC_2	21 (6-30~6-50)	44~50
BC_2	19 (6-51~6-69)	53~55, 56~60, 62~64, 65~66, 68
BC_3	21 (6-30~6-50)	30~36, 39, 40, 48~50
BC_3	19 (6-51~6-69)	52, 54, 59, 61, 62, 64, 67, 68
BC_4	15 (1-63~1-77)	64, 65, 70, 73, 74, 77
BC_4	16 (2-7~2-22)	11, 12, 17, 20, 22
BC_4	11 (2-23~2-33)	24, 25, 26, 27,32
BC_4F_2	21 (4-51~4-71)	53, 56, 57, 60, 63~66, 69~71
BC_4F_2	19 (4-72~4-90)	72, 73, 75, 76, 79, 82, 83, 87
BC_4F_2	19 (4-91~5-15)	4-91, 4-92, 4-94, 5-1, 5-2, 5-5~5-15
BC_4F_2	19 (5-16~5-34)	18~20, 23, 26, 27, 29, 33, 34
BC_4F_2	14 (5-35~5-48)	35, 38, 40, 41, 46
BC_4F_2	21 (5-49~5-69)	50, 52, 53, 57, 60, 62, 63, 66
BC_4F_2	25 (5-70~5-94)	70~72, 77, 78, 83, 84, 85, 88, 93

Table S3. Result statistics of background selection

Population	Individual plants	L	X(g)	PRPG (PRPG= [L+X (g)]/2L*100%)
BC_2	153-1	86	34	69.77%
BC_2	153-2	86	45	76.16%
BC_2	153-3	86	52	80.23%
BC_2	153-4	86	44	75.58%
BC_2	153-5	86	57	83.14%
BC_2	153-6	86	39	72.67%
BC_2	154-1	86	52	80.23%
BC_2	153-2	86	45	76.16%
BC_3	CJ49-1	223	151	83.90%
BC_3	CJ49-2	223	157	85.20%
BC_3	CJ51-1	223	172	88.60%
BC_4	1-64	60	59	99.17%
BC_4	1-65	60	58	98.33%
BC_4	1-70	60	56	96.67%
BC_4	1-73	60	42	85.00%
BC_4	1-74	60	56	96.67%
BC_4	1-77	60	59	99.17%
BC_4	2-11	60	59	99.17%
BC_4	2-12	60	5	54.17%
BC_4	2-20	60	59	99.17%
BC_4	2-22	60	54	95.00%
BC_4	2-24	60	59	99.17%
BC_4	2-25	60	56	96.67%
BC_4	2-26	60	59	99.17%
BC_4	2-27	60	54	99.17%
BC_4	2-32	60	59	99.17%

Table S4. Primers used in background selection

Primer	Sequences
EF	CTCGTAGACTGCGTACC
ER	CTGACGCATGGTTAA
MF	GACGATGAGTCCTGA
MR	TACTCAGGACTCAT
SAF	CTCGTAGACTGCGTACAAGCT
SAR	TGTACGCAGTCTAC
Taq I-F	GACGATGAGTCTGTCA
Taq I-R	CGTGACAGACTCAT
PF	CTCGTAGACTGCGTACATGCA
PR	TGTACGCAGTCTAC
EA	GACTGCGTACCAATTCA
EC	GACTGCGTACGAATT
MG	GATGAGTCCTGAGTAAG
MC	GATGAGTCCTGAGTAAC
SA	GACTGCGTACAAGCTCA
MC	GATGAGTCCTGAGTAAC
MG	GATGAGTCCTGAGTAAG
P0	GACTGCGTACATGCAG
TC	GATGAGTCTGTCACGAC
TG	GATGAGTCTGTCACGAG

Table S5. Reaction systems for PCR amplifications

Component	Volume（μL）
DNA	2.0
Primer-1	0.3
Primer-2	0.3
Buffer（10x）	1.5
Mg^{2+}（25 mM）	1.2
dNTPs（10 mM）	0.2
Taq（5 U/μL）	0.15
ddH_2O	9.35
Total	15.0
